# Clinical observation of low temperature plasma ablation combined with collagenase injection in lumbar disc herniation

**DOI:** 10.1097/MD.0000000000039739

**Published:** 2024-09-20

**Authors:** Yulin Zhang, Qifan Fang, Yongming Zhao, Ying Yang

**Affiliations:** a Department of Pain, Nanchong Central Hospital (Nanchong Hospital, Beijing Anzhen Hospital)/The Second Clinical School of North Sichuan Medical College, Nanchong, Sichuan, China.

**Keywords:** collagen enzyme, lumbar disc herniation, plasma ablation

## Abstract

To observe the effect of low temperature plasma ablation combined with collagenase injection on lumbar disc herniation. 90 patients with lumbar disc herniation admitted to the pain department of our hospital and receiving surgical treatment from April 2021 to April 2023 were included in this retrospective study, and were divided into 2 groups according to different treatment plans. One group was treated with low-temperature plasma ablation combined with collagenase injection, and the other group was treated with low-temperature plasma ablation alone. The sample size of both groups was 80 cases. Peripheral blood was collected by fasting in the morning at 5 time points before surgery, 1, 3, 7 and 14 days after surgery, and 5 mL of whole venous blood was collected by disposable vacuum blood collection device. Serum levels of pro-inflammatory factor interleukin (IL)-1α, IL-1β, IL-6, IL-8, tumor necrosis factor-α and anti-inflammatory factor IL-4, IL-10 were detected by ELISA. VAS scores were used to evaluate postoperative low back pain. ODI and Lehmann Lumbar Function Scale were used to evaluate postoperative lumbar function. The contents of IL-1α, IL-1β, IL-6, IL-8, tumor necrosis factor-α and anti-inflammatory factor IL-4, IL-10 in the cryo-plasma ablation combined with collagenase injection group were significantly lower than those in the cryo-plasma ablation group alone (*P* < .05). The VAS score of cryo-plasma ablation combined with collagenase injection group was significantly lower than that of cryo-plasma ablation group at 1 day and 3 months after treatment, and the difference was statistically significant (*P* < .05). The ODI score of cryo-plasma ablation combined with collagenase injection group was significantly lower than that of cryo-plasma ablation group at 1 day and 3 months after treatment, and the difference was statistically significant (*P* < .05). The Lehmann score of cryo-plasma ablation combined with collagenase injection group was significantly higher than that of cryo-plasma ablation group at 1 day and 3 months after treatment, and the difference was statistically significant (*P* < .05). The overall efficacy of low-temperature plasma ablation combined with collagenase injection is better than that of low-temperature plasma ablation alone. Low temperature plasma ablation combined with collagenase injection in the treatment of patients with lumbar disc herniation has less pain, faster recovery.

## 1. Introduction

Lumbar disk herniation (LDH) is a common and frequent clinical disease, and also one of the common causes of lumbar and leg pain. It is mainly caused by various reasons to promote the degeneration or rupture of the lumbar disc annulus fibrosus after injury. Under the action of external force, the nucleus pulposus protrudes from the weak or ruptured annulus fibrosus. Compression of adjacent spinal nerve roots causes low back pain and radiation-induced lower limb pain.^[1]^ Plasma ablation generally refers to low-temperature plasma radiofrequency ablation, and lumbar disc herniation refers to lumbar disc herniation, which can be treated by low-temperature plasma radiofrequency ablation.^[2]^ From the present point of view, low temperature plasma radiofrequency ablation for lumbar disc herniation has a relatively good effect, the clinical effective rate is 90% or even above. However, in order to achieve a better clinical treatment effect, the corresponding indications should be met. Low-temperature plasma radiofrequency ablation is a minimally invasive surgery, which is suitable for patients with inclusive lumbar disc herniation, which can be manifested as simple low back pain, simple lower limb radiation pain, or patients with both low back pain and leg pain. Collagenase chemolysis, also known as collagenase nucleolysis. Under the guidance of C-arm X-ray machine and CT, collagenase is accurately injected into and around the herniated intervertebral disc to dissolve and absorb the herniated intervertebral disc and relieve its compression on the nerve root, achieving the same effect as surgical removal of the herniated intervertebral disc. The excellent and good rate can reach more than 90%. This treatment has become one of the effective minimally invasive interventional treatment methods for cervical spondylosis and lumbar disc herniation caused by disc herniation because of its small trauma, few complications and reliable efficacy.^[3,4]^ Therefore, the purpose of this study was to observe the efficacy of LTC in the treatment of lumbar disc herniation in order to find a better treatment method for LDH.

## 2. Materials and methods

### 2.1. Research objects

This study was approved by the Nanchong Central Hospital ethics committee, the research into all of the patients with intervertebral disc herniation were in April 2021 to April 2023 in our hospital during the period of pain, hospitalization and surgical treatment of 160 cases of patients with lumbar disc as observation objects. All subjects were screened in detail against the inclusion and exclusion criteria of this study, and unqualified subjects were excluded to provide qualified samples for this study. Inclusion criteria: patients who met the diagnosis of lumbar disc herniation by clinical symptoms and CT or MRI imaging examination, and met the diagnostic criteria of LDH in the guidelines for grading diagnosis and treatment of lumbar disc herniation, and required surgical treatment, patients with leg pain more severe than back pain, with paresthesia in dermatomal distribution; For the first time, the patient has not received corresponding treatment, including conservative treatment, surgical treatment or traditional Chinese medicine treatment; The patient had obvious radicular pain; Magnetic resonance imaging (MRI) confirms segmental disc herniation corresponding to leg pain or secondary non-severe spinal canal and lateral recessed stenosis. Exclusion criteria: acute or chronic inflammation such as osteoarthritis and ankylosing spondylitis; Patients with immune system diseases such as SLE and rheumatoid arthritis; Taking immunosuppressive drugs; had undergone surgery for LDH or had a history of vertebral surgery; Patients with serious organ dysfunction such as heart, liver and kidney, hypertension or diabetes; Patients with blood diseases, serious infectious diseases or malignant tumors. The patients had decreased muscle strength and sellar lesions.

### 2.2. Group and treatment plan

They were divided into 2 groups according to the different treatment regimens: one group was cryo-plasma ablation combined with collagenase injection, and the other group was cryo-plasma ablation alone.

Low-temperature plasma ablation is a combination of thermal coagulation and ablation to remove part of the nucleus pulposus. Low-temperature plasma ablation technology is used to vaporize part of the nucleus pulposus tissue in real time to reduce the volume of the nucleus pulposus. Then, the nucleus pulposus tissue contacted by the knife head was warmed to about 70 degrees by accurate thermal shrinkage technology, which reduced the total volume of the nucleus pulposus and reduced the pressure in the intervertebral disc, so as to achieve the purpose of decompression and treatment.

Collagenase lysis is to inject collagenase into the diseased intervertebral disc or around the protrusion, and rely on the pharmacological action of collagenase to decompose collagen fibers to dissolve the collagen tissue, so as to reduce or disappear the protrusion, so as to relieve or eliminate its compression on the nerve tissue, so as to improve the clinical symptoms of patients. Collagenase is a major soluble collagen enzyme, can effectively dissolve in the nucleus pulposus and fiber ring I and II collagen, and human tissue osmotic pressure equal collagenase solution does not destroy tissue cells and nerve cells, the protein hemoglobin, cheese, keratin protein such as sulfate without damage, can decompose under normal physiological environment and the ph value of collagen fibers, It is degraded to the relevant amino acids and absorbed by the plasma. The collagenase injection or inside of the pathological changes of intervertebral disc protrusion, relying on the pharmacological effects of collagenase decomposition of collagen fibrils to dissolve collagen tissue, reduce protrusions or disappear, to alleviate or eliminate its repression of nerve tissue, so that the patient’s clinical symptoms improved, this treatment is called collagenase dissolved art. It also brings complications. The process of collagen dissolution and hydrolysis following collagenase chemolysis may lead to increased pressure within the intervertebral disc and spinal canal, exacerbating compression on the nerve roots. This can intensify lower back and leg pain and even lead to serious complications such as nerve root damage. The incidence of these complications may be proportional to the dosage of collagenase used.

The basic information of all participants was collected, including: name, age, education level, course of disease, weight, height, smoking history, drinking history, family history, chronic disease diagnosis, spinal surgery history and other clinical factors. There was no significant difference in the general data between the 2 groups (*P* < .05; see Table 1).

**Table 1:** Comparison of general data between the 2 groups.

Group	n	Age (yr)	Gender (male/female)	Duration of disease (yr)	Vertebral disease (L2-3/L5-S1/L4-5/L3-4)	Body mass index (kg/m^2^)
Low temperature plasma ablation combined with collagenase injection group	80	70.25 ± 8.25	43/37	7.24 ± 2.12	11/22/29/18	24.49 ± 3.82
Low temperature plasma ablation group	80	69.23 ± 9.20	44/36	7.42 ± 1.92	10/26/27/17	24.62 ± 3.74
χ^2^*/Z/t*		0.690	0.092	0.287	1.827	0.123
*P*		.455	.905	.769	.392	.883

#### 2.2.1. LT group

Using the posterolateral approach on the affected side, puncture was performed layer by layer under CT guidance until reaching the posterior one-third of the lesioned intervertebral disc (multiple CT scans were performed during the procedure to avoid damage and unforeseen events). Subsequently, a fixed adhesive sleeve was applied to stabilize the puncture needle and prevent its displacement. A small amount of physiological saline was injected into the puncture needle as a plasma carrier, and CT scans were performed to confirm the position of the plasma knife tip in the center of the nucleus pulposus of the intervertebral disc. Once the position was satisfactory and no discomfort was reported during testing, the plasma machine power was set to level 2, with 9 seconds of coagulation followed by 9 seconds of ablation. If the patient developed neurological symptoms, the procedure was immediately stopped. The needle tip position was adjusted accordingly; if no symptoms occurred, the procedure continued, repeating the coagulation and ablation 3 times. After confirming the patient’s comfort, the plasma machine power was increased to level 3, and the procedure was repeated 3 times with 9 seconds of coagulation followed by 9 seconds of ablation. Throughout the entire coagulation and ablation process, communication with the patient was maintained, and careful observation of the patient’s consciousness, vital signs, pain changes, and lower limb neurological function was conducted.

#### 2.2.2. LTC group

**Figure 1.**
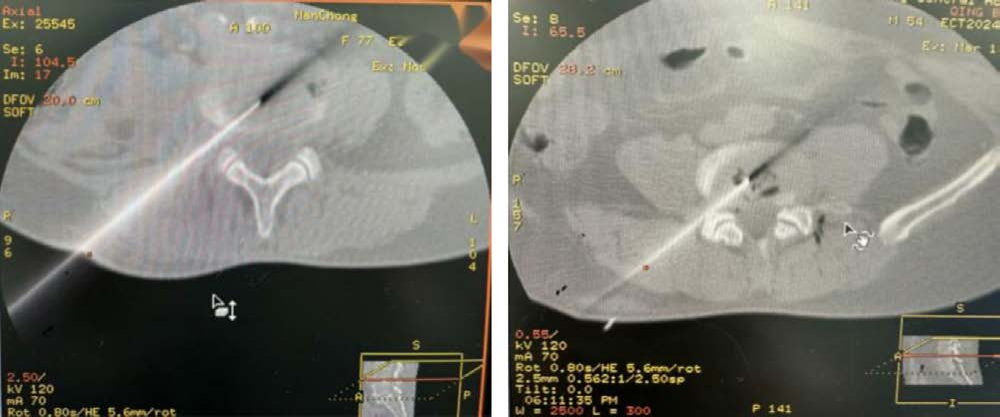
The puncture process and the working area of the plasma ablation blade.

The plasma operation and energy usage were entirely identical. After low temperature plasma ablation, a No. 7 needle (8–10 cm long) was inserted through the medial aspect of the facet joint under radiographic guidance, entering the widest part of the intervertebral disc space, and the needle angle was adjusted appropriately based on CT scan results. After penetrating the ligamentum flavum and reaching the extradural anterior space, the needle tip was adjusted to approach the surface of the protrusion. After confirming the absence of blood or cerebrospinal fluid upon withdrawal, 60 mg of 2% lidocaine plus 5 mg of dexamethasone were injected for a local anesthetic test. Observation was conducted for 15–20 minutes, and if no spinal anesthesia occurred and the determined anesthesia level corresponded to the distribution area of the nerve root on the same side, 600 U of collagenase were injected (Fig. 1).

### 2.3. Detection of inflammatory factors in serum samples

The peripheral blood samples of LDH patients in the 2 groups were collected by fasting in the morning at 5 time points before operation,7 days after operation, and 5ml of whole venous blood was collected by disposable vacuum blood collector. The levels of proinflammatory factors interleukin (IL)-1α, IL-1β, IL-6, IL-8, tumor necrosis factor (TNF)-α and anti-inflammatory factor IL-4, IL-10 in blood were determined by ELISA.

### 2.4. Judgment criteria for clinical efficacy

In this study, the Modified MacNab Criteria were used to evaluate the clinical efficacy of LDH patients in the 2 groups. The specific evaluation Criteria were as follows: Excellent: patients with preoperative low back pain completely disappeared, no motor function limitation, and returned to normal work, life and daily activities; Good: the patient still has mild preoperative low back pain symptoms, occasional pain, activity is slightly limited but has no impact on work, life and daily activities; Fair: the patient’s back pain symptoms were relieved before operation, and there was still pain, and the activity was slightly limited, which had a slight impact on work, life and daily activities; Poor: the patient’s preoperative low back pain symptoms did not alleviate, even more aggravated, nerve compression performance, need further surgery.

### 2.5. Postoperative lumbar function indicators

The function indicators included Visual Analog Scale (VAS) score to understand the improvement of postoperative low back pain, Oswestry Disability Index (ODI) and Lehmann Lumbar function Scale to evaluate postoperative lumbar function. The differences of above indexes between the 2 groups were compared.

### 2.6. Statistical analysis and result arrangement

The experimental results and corresponding experimental data obtained from the experiment were sorted out and analyzed by SPSS20.0 statistical software. The measurement data were tested for normal distribution and homogeneity of variance. Data conforming to normal distribution and homogeneity of variance were expressed as mean ± standard deviation, and group t test was used for comparison between the 2 groups. Enumeration data were expressed as percentage and χ^2^ test was performed. Dunnett’s T3 test was used when the variance was uneven. α = 0.05 was used to indicate that the difference was statistically significant.

## 3. Results

### 3.1. Comparison of the levels of inflammatory factors in serum samples of LDH patients in different periods after operation between the 2 groups

The contents of IL-1α, IL-1β, IL-6, IL-8, TNF-α and anti-inflammatory factor IL-4, IL-10 in the LTC group were significantly lower than those in the LT group alone, and the differences were statistically significant (*P* < .05). Are shown in Table 2.

**Table 2:** Comparison of inflammatory factors in serum samples of LDH patients in 7 days after operation between the 2 groups (pg/mL, $\bar{x}$ ± s).

Group	TNF-α	IL-1α	IL-1β	IL-6	IL-8	IL-4	IL-10
Low temperature plasma ablation combined with collagenase injection group	12.22 ± 3.56	62.63 ± 24.62	47.22 ± 8.98	432.62 ± 36.54	302.55 ± 73.82	195.84 ± 28.62	164.49 ± 38.33
Low temperature plasma ablation group	20.98 ± 3.72	81.90 ± 30.63	65.53 ± 12.62	605.32 ± 64.43	472.73 ± 102.33	262.58 ± 43.23	324.92 ± 44.74
*t*	6.632	5.732	7.634	10.938	12.284	5.357	19.737
*P*	<.001	<.001	<.001	<.001	<.001	<.001	<.001

IL = interleukin, LDH = Lumbar disk herniation, TNF = tumor necrosis factor.

### 3.2. Comparison of VAS score, ODI score and Lehmann lumbar function score between the 2 groups after LDH

There was no significant difference in VAS score between LTC group and LT group before operation (*P* > .05). VAS score of LTC group and LT group after treatment for 1 day and 3 months was significantly lower than that before operation. The VAS score of the LTC was significantly lower than that of the LT group at 1 day and 3 months after treatment, and the difference was statistically significant (*P* < .05), as shown in Table 3.

**Table 3:** Comparison of postoperative VAS scores between the 2 groups of LDH patients (point, $\bar{x}$ ± s).

Group	Preoperative	1 d after surgery	3 months after surgery
Low temperature plasma ablation combined with collagenase injection group	6.41 ± 0.76	3.65 ± 0.44*	1.95 ± 0.28*
Low temperature plasma ablation group	6.45 ± 0.78	2.52 ± 0.37*	1.14 ± 0.24*
* t*	0.260	14.615	16.817
* P*	.879	<.001	<.001

LDH = lumbar disk herniation, VAS = Visual Analog Scale.

Compared with that before operation,

* *P* < .05.

There was no significant difference in preoperative ODI scores between the LTC group and the LT group (*P* > .05). The ODI scores of the LCT group and the CT group were significantly lower than those 3 months after treatment. The ODI score of the LCT group was significantly lower than that of the LT group at 3 months after treatment, and the difference was statistically significant (*P* < .05), as shown in Table 4.

**Table 4:** Comparison of postoperative ODI scores between the 2 groups of LDH patients (point, $\bar{x}$ ± s).

Group	Preoperative	1 d after surgery	3 months after surgery
Low temperature plasma ablation combined with collagenase injection group	56.21 ± 5.72	47.03 ± 4.88*	32.05 ± 3.35*
Low temperature plasma ablation group	56.54 ± 5.03	41.68 ± 4.44*	26.14 ± 2.74*
* t*	0.237	5.848	7.565
* P*	.890	<.001	<.001

LDH = lumbar disk herniation, ODI = Oswestry Disability Index.

There was no significant difference in Lehmann score between LCT group and LT group before operation (*P* > .05). The Lehmann score of LCT group and LT group after treatment for 1 day and 3 months was significantly higher than that before operation. The Lehmann score of the LTC group after treatment for 1 day and 3 months was significantly higher than that of the LT group alone (*P* < .05), as shown in Table 5.

**Table 5:** Comparison of Lehmann scores between the 2 groups of LDH patients after surgery (point, $\bar{x}$ ± s).

Group	Preoperative	1 d after surgery	3 months after surgery
Low temperature plasma ablation combined with collagenase injection group	27 ± 5	45 ± 8*	63 ± 13*
Low temperature plasma ablation group	28 ± 5	62 ± 14*	74 ± 14*
* t*	0.201	8.546	4.527
* P*	.905	<.001	<.001

LDH = Lumbar disk herniation

### 3.3. Comparison of clinical efficacy between the 2 groups

There was no significant difference in clinical efficacy between LCT group and LT group alone (*P* > .05). As shown in Table 6.

**Table 6:** Comparison of clinical efficacy between the 2 groups.

Group	Great	Good	Fair	Poor
Low temperature plasma ablation combined with collagenase injection group	15	19	10	1
Low temperature plasma ablation group	14	18	11	2
χ^2^	3.348			
* P*	.249			

## 4. Discussion

Intervertebral disc degeneration is the main cause of LDH. However, the process of intervertebral disc degeneration is not only reflected in the changes of intervertebral disc tissue morphology, but also accompanied by significant changes in the biochemical indicators of intervertebral disc tissue, including the decreased contents of proteoglycan and water in nucleus pulposus tissue, the change of collagen type, the decreased expression of immune substances and growth factors, etc.^[5–7]^ Related studies on these biochemical indicators have shown^[8]^ that changes in biochemical indicators during disc degeneration may be closely related to validation responses. In recent years, clinical studies have confirmed^[9]^ that the degree of inflammatory reaction and the level of inflammatory factors are correlated with the pathological process of intervertebral disc degeneration. The effects of inflammatory factors on LDH are mainly reflected in 3 aspects, including synthesis and decomposition of intervertebral disc matrix, degree of inflammatory reaction and apoptosis of intervertebral disc cells.^[10]^ Studies have shown^[11]^ that inflammatory factors such as IL-1β, IL-6 and TNF-α are highly expressed in the degenerative nucleus pulposus and annulus fibrosus, and can promote the production and secretion of a variety of matrix metalloproteinases (MMPs) in nucleus pulposus and annulus fibrosus, leading to cartilage matrix degradation. At the same time, these inflammatory factors can inhibit the synthesis of proteoglycans and type II collagen by chondrocytes, promote the production of type I collagen, and eventually cause the degeneration and death of chondrocytes. Therefore, the level of inflammatory factors, especially the expression level of proinflammatory factors, is closely related to the condition of LDH patients.^[12]^ The degenerative intervertebral disc tissue in patients with LDH has great changes in character and composition, which can induce the body to produce various inflammatory factors, and then up-regulate the level of MMPs in intervertebral disc tissue, affect matrix metabolism, and cause intervertebral disc herniation. However, after disc herniation, it can in turn stimulate the body to produce more inflammatory factors, thus further aggravating disc degeneration.^[13]^ Inflammatory factors can be divided into proinflammatory factors and anti-inflammatory factors according to their different regulatory effects on inflammatory response. Among them, IL-1, IL-6, IL-8 and TNF-α are considered as proinflammatory factors, while IL-2, IL-4 and IL-10 are considered as anti-inflammatory factors.^[14]^ The inflammatory factors that are closely related to intervertebral disc degeneration are mainly IL-1α, IL-1β, IL-6, IL-8 and TNF-α.^[15,16]^

Indications for low-temperature plasma ablation for lumbar disc herniation include disc herniation, symptoms of nerve roots in one leg such as inability to walk far, pain and numbness in 1 leg, and incomplete detachment of the nucleus pulposus, including the disc. If the nucleus pulposus is free or detached, it is not suitable. Treatment requires minimally invasive foraminal endoscopy or disc endoscopy or open surgery. In the past, the first generation plasma had only 1 ablation target, but now the second generation plasma can ablate multiple targets, which greatly reduces the patient’s symptoms. The operation was safe and painless. Patients will feel relieved immediately during surgery, and the probability of reoccurrence will be greatly reduced, which will bring very safe treatment effect to patients and achieve better clinical satisfaction. This treatment of inclusive disc herniation and intractable pain in the lower lumbar spine is primarily targeted for these 2 indications. For patients with pathogenic section comparatively clear, by CT, MRI and other imaging examination, clear the location of the lumbar disc to focus on a side, near the central type is ideal surgical indications, some patients are diffuse and herniation, in this part of the patients to careful evaluation of symptoms and pathogenesis of lumbar consistency. If the choice of responsible lumbar disc is clear, the effect of low temperature plasma radiofrequency ablation is better. For patients with partial lumbar spondylolisthesis, generalized spinal stenosis, and ineffective conservative treatment, and even patients with serious neurological symptoms, such as leg and hand numbness, the effect of low-temperature plasma radiofrequency ablation will be reduced. Therefore, this operation should be carefully selected. At the same time, according to the patient’s wishes, the doctor’s surgical skills, and the imaging characteristics of the disease, whether to carry out low-temperature plasma radiofrequency ablation. Patients with lumbar disc herniation usually develop after lumbar sprain or fatigue, such as increased abdominal pressure, inappropriate waist posture, and sudden weight bearing, pregnancy, etc. Patients in daily life should pay attention to sleep hard bed, pay attention to waist warm measures, avoid fatigue, ensure the correct posture and sitting posture. When pain is relieved, appropriate activities such as lumbar and back muscle exercise can be carried out, which is conducive to the relief of lumbar disc herniation.^[17–20]^ The advantages of minimally invasive intervention are as follows: lumbar disc herniation is a common and frequent clinical disease, which seriously harms the physical and mental health of patients, and is also 1 of the main causes of low back and leg pain. Its conservative treatment course is long, and the long-term effect is uncertain; The open operation has great damage and more complications. In contrast, minimally invasive treatment of lumbar disc herniation has obvious advantages. Low temperature plasma and collagenase lysis are 2 minimally invasive treatment methods that have been widely recognized in clinical practice. They do not operate, have little trauma, protect the annulus fibrosus wall to the maximum extent, and do not damage the normal intervertebral disc tissue. There was no damage to the bone structure and almost no effect on the stability of the spine. The rate of intervertebral disc re-herniation was low. After operation, the disc is reduced in size, which does not affect the appearance. The nerve root interference is small, thus reducing the tissue damage, and can greatly reduce the patient’s pain and shorten the recovery cycle.

Collagenase, chemically known as Collagenase, can specifically hydrolyze the 3-dimensional helical structure of natural collagen at physiological PH and temperature without damaging other proteins and tissues. The chemical nature of collagenase is a kind of protein. Therefore, it is very sensitive to temperature, PH and various factors leading to protein denaturation, and is easily affected by external conditions to change its conformation and properties. Collagenase can be divided into human endogenous collagenase and medicinal collagenase according to their different ways of existence. Endogenous collagenase in human body refers to the collagenase in the human body itself, such as gingival tissue, haptotheca and other epithelial tissues, joint synovium and intervertebral disc exist in varying degrees, and it plays an indispensable role in the decomposition process of collagen in the body. Medicinal collagenase is a white or white sterile lyophilized powder needle biological agent extracted, purified and refined from the fermentation broth of Clostridium histolytica by high-tech means of biopharmaceuticals. Collagenase is extracted from Clostridium tissue olyticus and mainly hydrolyzes collagen components in connective tissue. When the tissue to be digested is stiff and contains more connective tissue or collagen, the effect of trypsin to dissociate cells is poor. In this case, collagenase can be used to dissociate cells. Collagenase only had a digestive effect on the interstitium but had little effect on the epithelium. Therefore, it is suitable for digestion and separation of fibrous tissue, epithelium and cancer tissue, which can separate epithelial cells from collagen components without damage. The usual dose is a final concentration of 200 U/mL (approximately 1 mg/ mL) or 0.03% to 0.3%. Collagenase can be divided into types i, ii, iii, iv, v and special collagenase for hepatocytes, and the type of collagenase should be selected according to the tissue type to be separated and digested.^[21–23]^

Some studies conducted immunohistochemical tests on surgically resected intervertebral disc tissues, and found that the protruded or prolapse free intervertebral disc tissues not only had a large number of monocytes infiltrated around them, but also had significantly higher expression levels of inflammatory mediators such as IL-1, IL-6 and TNF-α.^[24]^ Moreover, recent studies have found^[25]^ that most cytokines have been confirmed to be secreted and produced by exocytic and free endothelial cells, bulging chondrocytes, histiocytes and fibroblasts. Immunohistochemical analysis of the degenerated intervertebral disc tissue showed that TNF-α, IL-1α, IL-1β, IL-6 and other inflammatory factors were expressed at high levels. Further studies confirmed that inflammatory factors were mainly produced by fibroblasts, prolapse and free endothelial cells and bulging chondrocytes. A clinical study also showed^[26]^ that the expression of IL-6 and IL-6 receptor in chondrocytes in the degenerative intervertebral disc tissues of LDH patients were positive, while the expression of IL-6 receptor in the intervertebral disc tissues of non-LDH control patients was negative. There were positive expressions of IL-8 and McP-1 in the degenerated intervertebral disc tissues of LDH patients with bulging type, protruding type and free prolapse type, and the expression levels of IL-8 and McP-1 in bulging type were lower than those in protruding type and free prolapse type.

Clinical studies have shown that the expression levels of IL-6 and IL-8 are higher in LDH patients with prominent symptoms of low back and leg pain, which suggests that IL-6 and IL-8 may be one of the important reasons for discogenic low back pain. A recent study confirmed^[27]^ that the expression levels of TNF-α and IL-6 in the degenerative disc tissues of LDH patients were positively correlated with the ODI score index, which indicated that the expression level of inflammatory mediators could effectively reflect the severity of low back and leg pain, and might be one of the important reasons for low back and leg pain caused by disc herniation. This also provides a new therapeutic target for clinical complete relief of clinical symptoms.^[28]^

In conclusion, for patients with lumbar disc herniation, the overall efficacy of low-temperature plasma ablation combined with collagenase injection is better than that of low-temperature plasma ablation alone. LTC in the treatment of patients with lumbar disc herniation has less pain, faster recovery and less severe immune stress response.

## Author contributions

**Conceptualization:** Yulin Zhang, Qifan Fang, Ying Yang.

**Data curation:** Yulin Zhang, Qifan Fang, Ying Yang.

**Formal analysis:** Yulin Zhang, Yongming Zhao, Qifan Fang.

**Investigation:** Yongming Zhao, Qifan Fang, Ying Yang.

**Methodology:** Yongming Zhao, Qifan Fang, Ying Yang.

**Supervision:** Yongming Zhao.

**Validation:** Ying Yang.

**Writing – original draft:** Yulin Zhang, Ying Yang.

**Writing – review & editing:** Yulin Zhang, Ying Yang.
